# An autopsy case of epignathus (immature teratoma of the soft palate) with intracranial extension but without brain invasion: case report and literature review

**DOI:** 10.1186/s13000-018-0776-y

**Published:** 2018-12-22

**Authors:** Mari Kirishima, Sohsuke Yamada, Mitsuhisa Shinya, Shun Onishi, Yuko Goto, Ikumi Kitazono, Tsubasa Hiraki, Michiyo Higashi, Akira I. Hida, Akihide Tanimoto

**Affiliations:** 1 Department of Pathology, Field of Oncology, Graduate School of Medical and Dental Sciences, Kagoshima University, 8-35-1 Sakuragaoka, Kagoshima, 890-8544 Japan; 2 Department of Pathology, Kagoshima University Hospital, 8-35-1 Sakuragaoka, Kagoshima, 890-8544 Japan; 3 Department of Pathology and Laboratory Medicine, Kanazawa Medical University, 1-1 Daigaku, Uchinada, Kahoku, Ishikawa 920-0293 Japan; 4 Department of Obstetrics and Gynecology, Kagoshima University Hospital, 8-35-1 Sakuragaoka, Kagoshima, 890-8544 Japan; 5 Department of Pediatric Surgery, Research Field in Medical and Health Sciences, Medical and Dental Area, Research and Education Assembly, Kagoshima University, 8-35-1 Sakuragaoka, Kagoshima, 890-8544 Japan

**Keywords:** Epignathus, Intracranial extension, Immature teratoma, Hypoxia

## Abstract

**Background:**

Epignathus is a rare congenital orofacial teratoma infrequently associated with intracranial extension. Intracranial extension of an epignathus indicates a poor prognosis; however, only a small number of such cases have been reported. While there have been some studies reporting cases of epignathus expanding directly into the cranium, others have reported no communication between an epignathus and an intracranial tumor.

**Case presentation:**

A fetus at gestational week 27 was suspected of having an epignathus with intracranial tumor as shown by ultrasonographic and magnetic resonance imaging. The fetus was stillborn and an autopsy was performed. An epignathus measuring 12 × 6 × 6 cm and weighing 270 g protruded from the mouth, with its base on the soft palate. An intracranial tumor weighing 14 g was located at the middle intracranial fossa and connected to the epignathus through the right side of the sella turcica. The intracranial tumor was encapsulated, and there was no invasion into the brain. Histologically, both the epignathus and intracranial tumor were immature teratomas, with neural and pulmonary components that were especially immature as compared to those of the internal organs and brain tissues of the fetus.

**Conclusion:**

There have been several reports of epignathus and intracranial tumors that did not communicate; therefore, careful evaluation is needed when a fetus is suspected of having an epignathus extending into an intracranial lesion. Our case supports the findings that an epignathus can directly expand into the cranium. Moreover, this is a rare case of an epignathus in which the intracranial lesion was encapsulated and did not invade the brain. These rare but important findings will provide additional, potential therapeutic strategies for gynecologists, neurosurgeons, and pathologists.

## Background

Here we report a prenatal case of an epignathus with intracranial extension, pathologically confirmed at autopsy. An epignathus is a rare congenital orofacial teratoma found in approximately 1:35,000–1:200,000 live births, accounting for 2–9% of all teratomas [1, 2]. In 6% of all the cases, teratomas are associated with malformations such as cleft palate, bifid tongue, and bifid uvula [1, 3]. Since epignathus fills the mouth and often protrudes outside, fatal airway obstruction frequently occurs. Prenatal detection allows an obstetrics team to schedule an elective cesarean delivery. Early intervention for ventilation is necessary, and immediate tracheostomy is often required. An epignathus is infrequently associated with an intracranial extension. Prenatal intracranial tumors include a variety of neoplasms, such as teratomas, neuroepithelial tumors, and craniopharyngiomas [4]. Prenatal intracranial teratomas have been reported to result in a poor prognosis [4, 5], and the clinical outcome of epignathus with intracranial extension remains unclear. Therefore, evaluating whether an epignathus extends into the cranium, whether the intracranial lesion and epignathus have different etiologies, and whether the intracranial tumor is invading the brain are crucial determinations in order to improve the prognosis of such cases.

## Case presentation

**Fig. 1:**
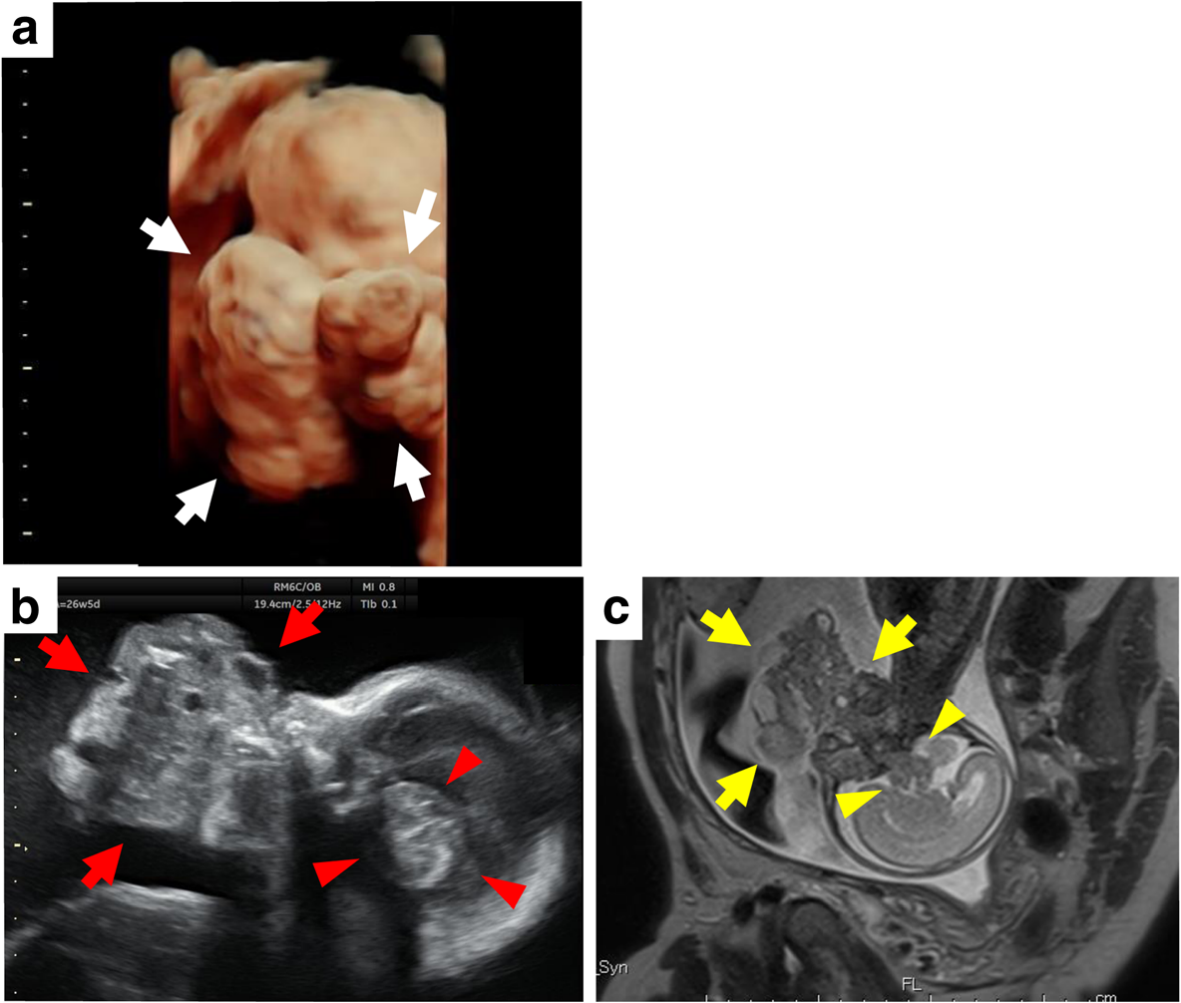
Ultrasonographic and MRI images. **a** Three-dimensional ultrasonographic image at gestational week 25 shows a giant tumor around the neck and face (white arrow), strongly suggesting an epignathus. **b** Ultrasonographic image at gestational week 26 shows a giant tumor around the neck (red arrow), and an intracranial tumorous lesion (red arrowhead). **c** MRI image at gestational week 25 suggests a connection between the giant tumor around the neck (yellow arrow) and the intracranial tumor (yellow arrowhead)

A 32-year-old woman, 3G2P (2 normal vaginal delivery, no abnormality) was referred to our hospital at 25 weeks of gestation for evaluation of a tumorous lesion located near the neck of the fetus, which was detected at gestational week 23. Prenatal three-dimensional ultrasonography performed at gestational week 25 showed a giant tumor around the neck and face (Fig. 1a). The spatial relationship between the tumor and the oral cavity was not confirmed; however, the possibility of an epignathus was strongly suspected. A prenatal ultrasound at gestational week 26 revealed a polyhydramnios, a giant tumor around the neck, and an intracranial tumorous lesion (Fig. 1b). Color Doppler ultrasonography showed arterial branches assumedly from the right internal carotid artery running into the giant tumor around the neck. Arterial branches from the right middle cerebral artery were suspected to be running into the intracranial lesion. These potential feeding arteries were of concern, because they could raise difficulties in the control of bleeding during an ex utero intrapartum treatment (EXIT) procedure. Magnetic resonance imaging (MRI) performed at gestational week 25 showed a giant tumor around the neck with heterogeneous iso-to-low signal density on T1-weighted images, and iso-to-high signal density on T2-weighted images. MRI also suggested a connection between the giant tumor and the intracranial lesion (Fig. 1c). An epignathus with intracranial extension was suspected. The possibility of preterm labor was a concern; therefore, supportive care with amnioreduction was to be provided until the fetus attained a certain weight and stage of organ development for performance of a cesarean delivery, at approximately 30 gestational weeks. At gestational week 27, after the amnioreduction was performed, a placental abruption occurred, and the fetus was delivered by emergency cesarean section. Chromosome analysis was not performed. The fetus was stillborn, and an autopsy was performed after 27 h, for pathological diagnosis and assessment of the intracranial lesion.

**Fig. 2:**
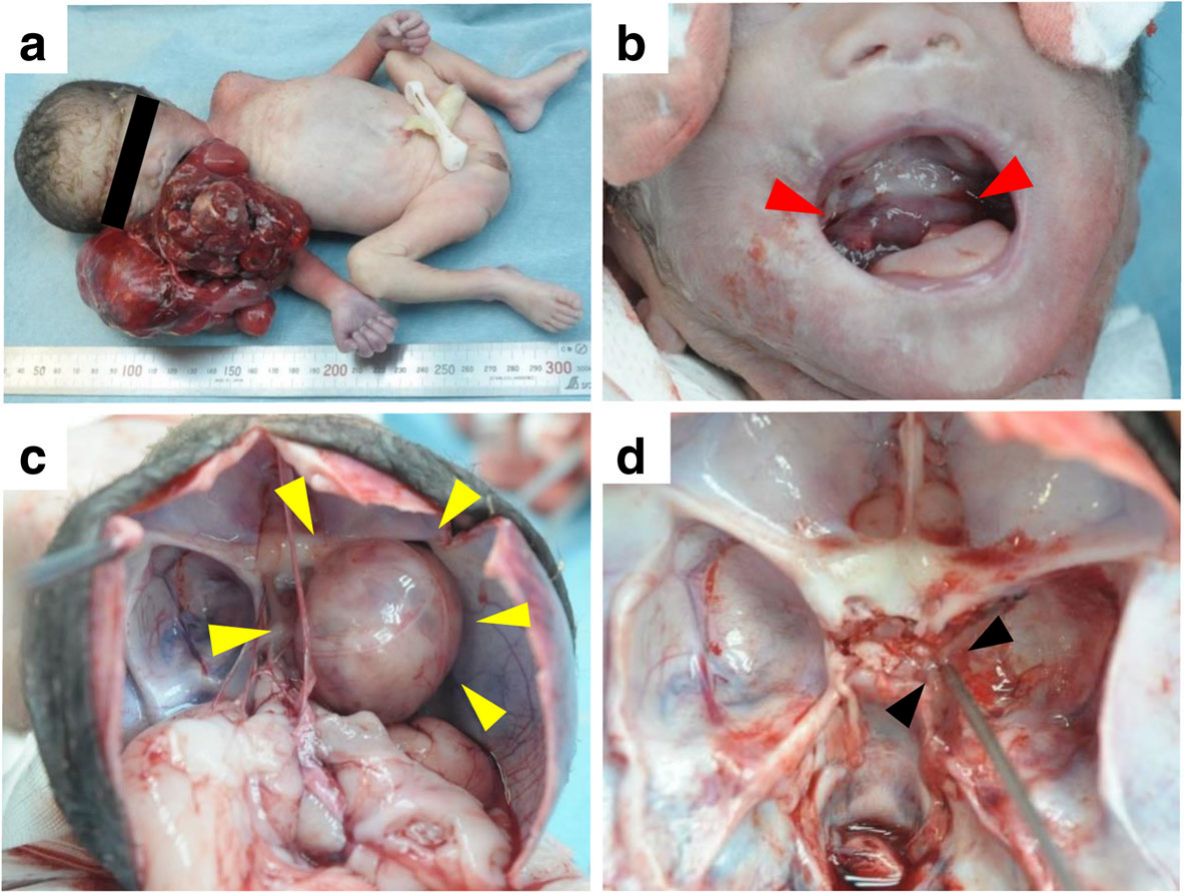
Macroscopic findings at autopsy. **a** The epignathus, a large reddish multicystic tumor is seen protruding from the mouth. **b** There is a defect in the soft palate within which the base of the epignathus is located (red arrowhead). **c** The intracranial tumor is covered by a thin capsule (yellow arrowhead) and there is no invasion of the brain. **d** After removal of the stem of the intracranial tumor, a small hole, measuring 2 mm in diameter, is detected on the right side of the sella turcica. A communication can be seen between the epignathus and the intracranial tumor through this hole (black arrowhead)

The fetus weighed 1228 g. At dissection, an epignathus was observed protruding from the mouth (Fig. 2a). It was a large, reddish, multicystic tumor measuring 12 × 6 × 6 cm and weighing 270 g. Neither a cleft palate nor cleft lips were found; however, there was a defect in the soft palate, within which the base of the epignathus was located (Fig. 2b). Upon opening the skull, an intracranial tumor weighing 14 g was detected in the middle cranial fossa. It was covered by a thin capsule, and there was no invasion into the brain (Fig. 2c). The tumor had a very short stem, 2 mm in diameter, which was located on the right side of the sella turcica. There was no tumor involvement in the optic foramen, foramen ovale, or optic nerve. After removal of the upper portion of the tumor above the stem, a small hole was found, measuring 2 mm in diameter, at the base of the sella turcica (Fig. 2d). To avoid facial and cranial deformation, in consideration of the distress of the bereaved family, we did not make further incisions through the bone, but rather, confirmed the continuance of the epignathus and intracranial tumor by using a sonde. The spreading of the epignathus into the cranium was consistent with the clinical ultrasonographic evaluation.

**Fig. 3:**
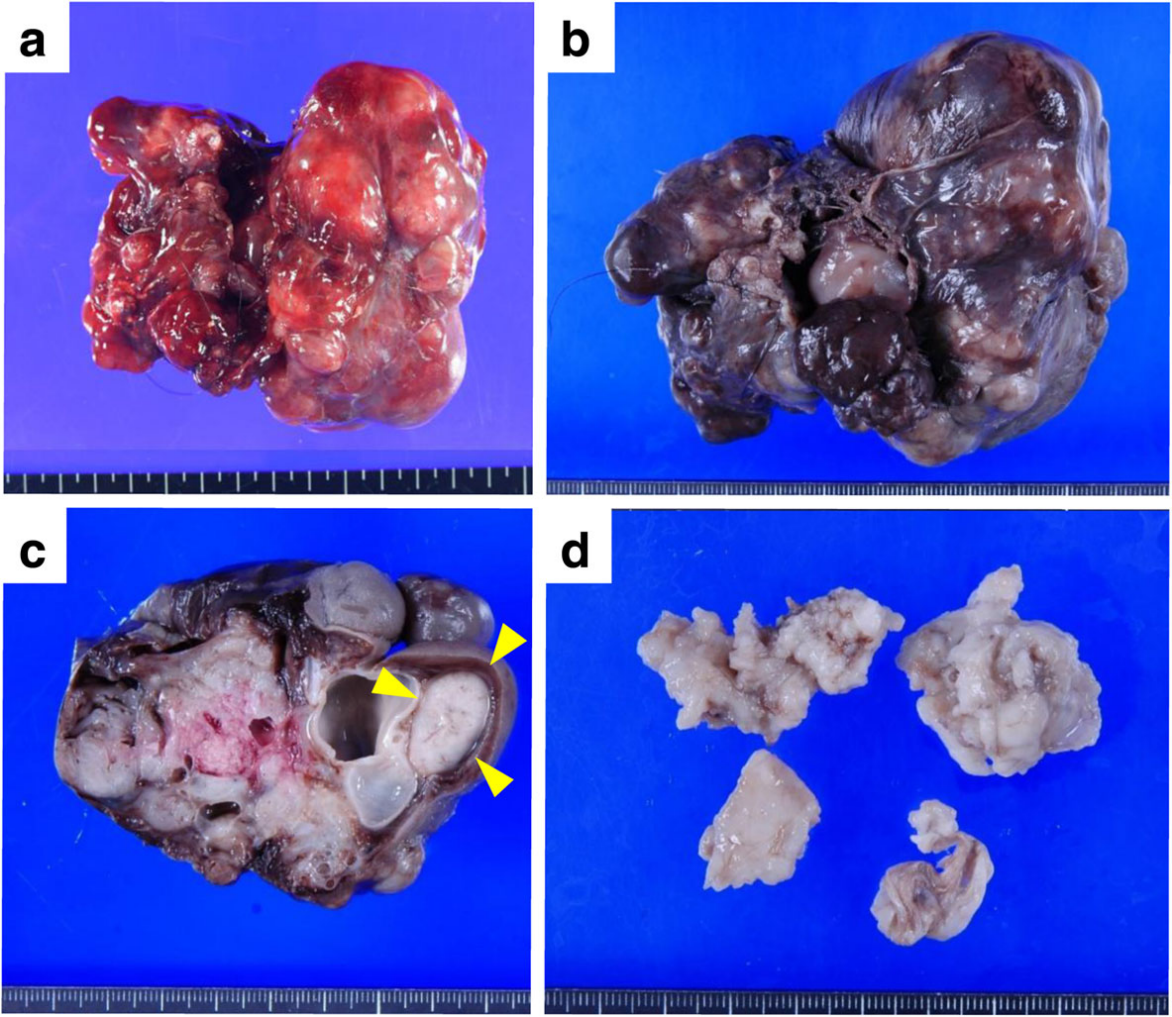
Macroscopic images of the epignathus. **a** At autopsy, the epignathus is a large reddish multicystic tumor. **b** After formalin fixation, the epignathus is seen as a dark-brown-colored tumor and cysts containing serous or bloody fluids. **c** The cut section shows whitish solid parts and cysts. A small component resembling a lung is seen (arrowhead). **d** The intracranial tumor is white and soft

**Fig. 4:**
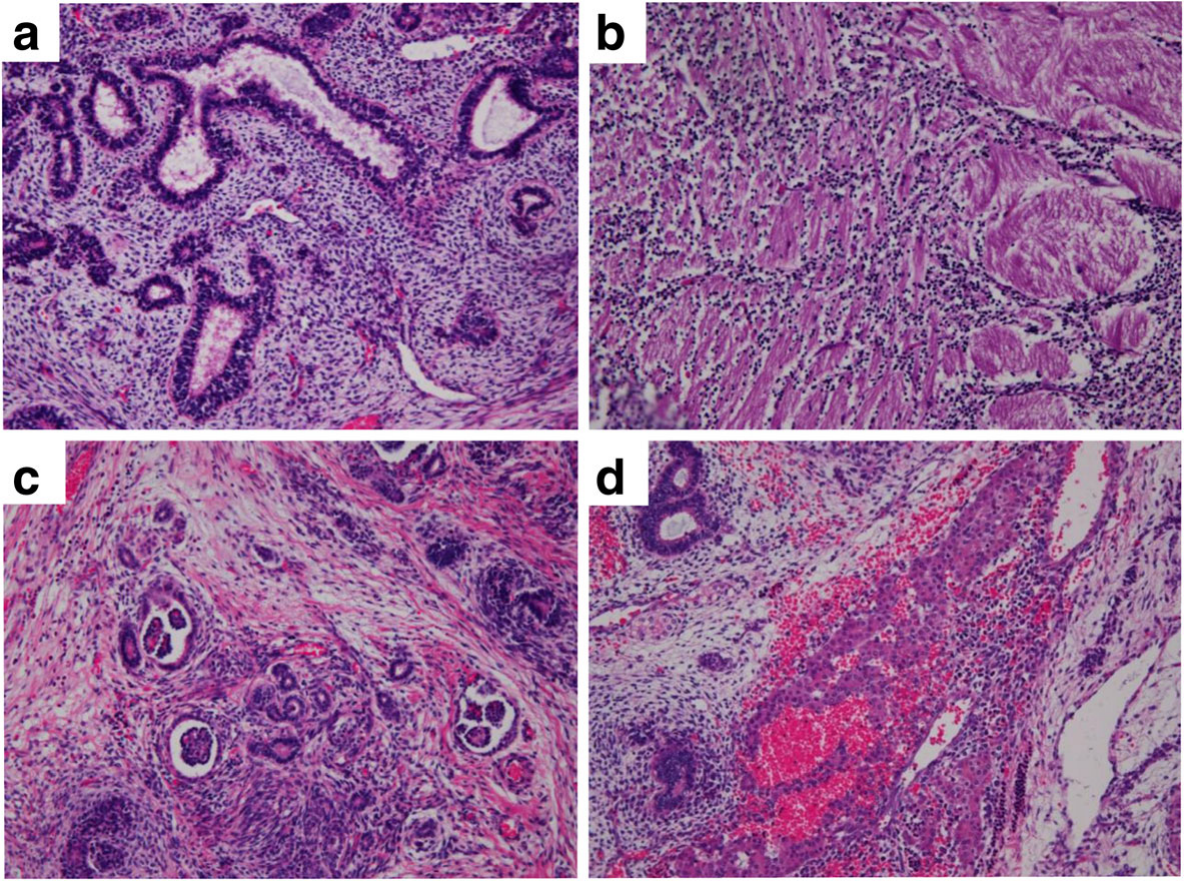
Microscopic images of tridermal components in the epignathus. **a** Immature neural tubules (hematoxylin and eosin [HE] stain, × 100). **b** Smooth muscle (HE stain, × 100). **c** Renal glomeruli (HE stain, × 100). **d** Hepatocytes (HE stain, × 100)

Macroscopically, the epignathus was a large multicystic tumor, containing white solid parts and cysts filled with serous or bloody fluid (Fig. 3a-c). Calcification was also seen. Microscopically, the prominent component of the tumor was immature neuroepithelial tissue (Fig. 4a). Immature neural tubules were seen, lined by dark hyperchromatic columnar cells with stratification and frequent mitoses, and were accompanied by glial tissue. Other components included melanocytes, retinal tissue, exocrine glands, skin, hepatocytes, renal glomeruli, lung tissue, glandular epithelium with goblet cells, squamous epithelium, cartilage, bone, adipose tissue, and smooth muscle (Fig. 4b-d).

**Fig. 5:**
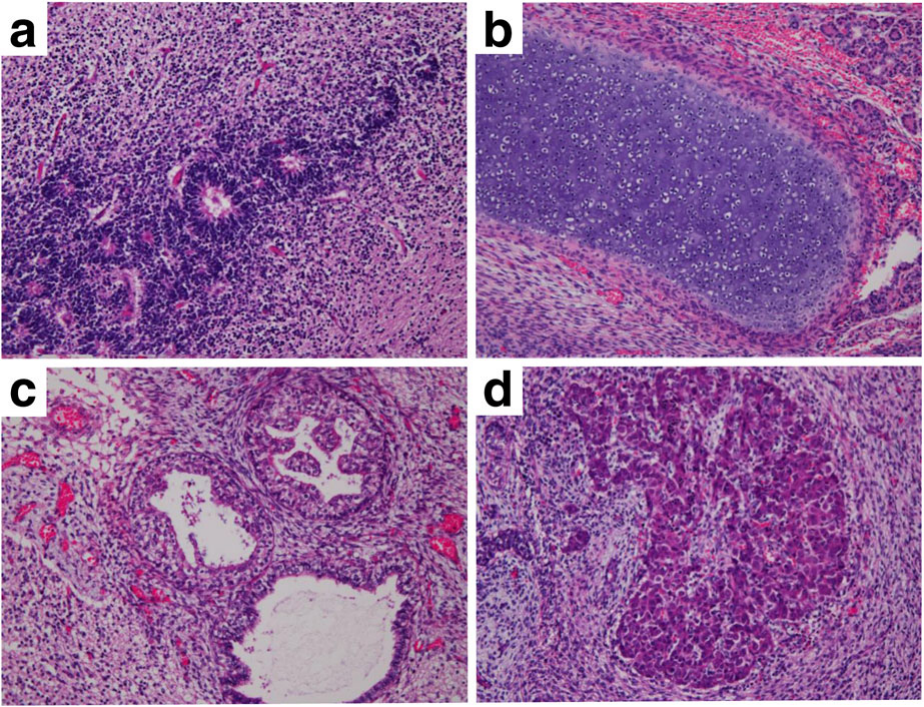
Microscopic images of tridermal components in the intracranial tumor. **a** Immature neural tubules (hematoxylin and eosin [HE] stain, × 100). **b** Cartilage (HE stain, × 100). **c** Esophagus (HE stain, × 100). **d** Hepatocytes (HE stain, × 100)

The intracranial tumor was macroscopically white and soft (Fig. 3d). The thin capsule was ruptured while we were excising the tumor at autopsy. Microscopically, the majority of the tumor was immature neuroepithelial tissue (Fig. 5a). The main components were immature neural tubules lined by dark hyperchromatic columnar cells with stratification and mitoses, and were accompanied by glial tissue. Other components included choroid plexus, aggregation of hepatocytes with extramedullary hematopoiesis, exocrine glands resembling pancreatic acini, columnar glandular cells resembling gastric foveolar epithelium, squamous epithelium, cartilage, and calcification (Fig. 5b-d).

The main components of the oral and intracranial tumors are summarized in Table 1.

**Table 1:** Components of the epignathus and the intracranial tumor

	epignathus	intracranial tumor
ectoderm	neural tube, glial tissue, retina, melanocytes, skin and appendages, exocrine glands (resemblings pancreatic tissue)	neural tube, glial tissue, choroid plexus
mesoderm	cartilage, bone, adipose tissue, smooth muscle, vessels	cartilage
endoderm	hepatocytes, renal glomeruli, lung, esophagus, gastric mucosa, intestinal mucosa with goblet cells	liver, glandular epithelium, exocrine glands (resembling pancreatic tissue)

**Fig. 6:**
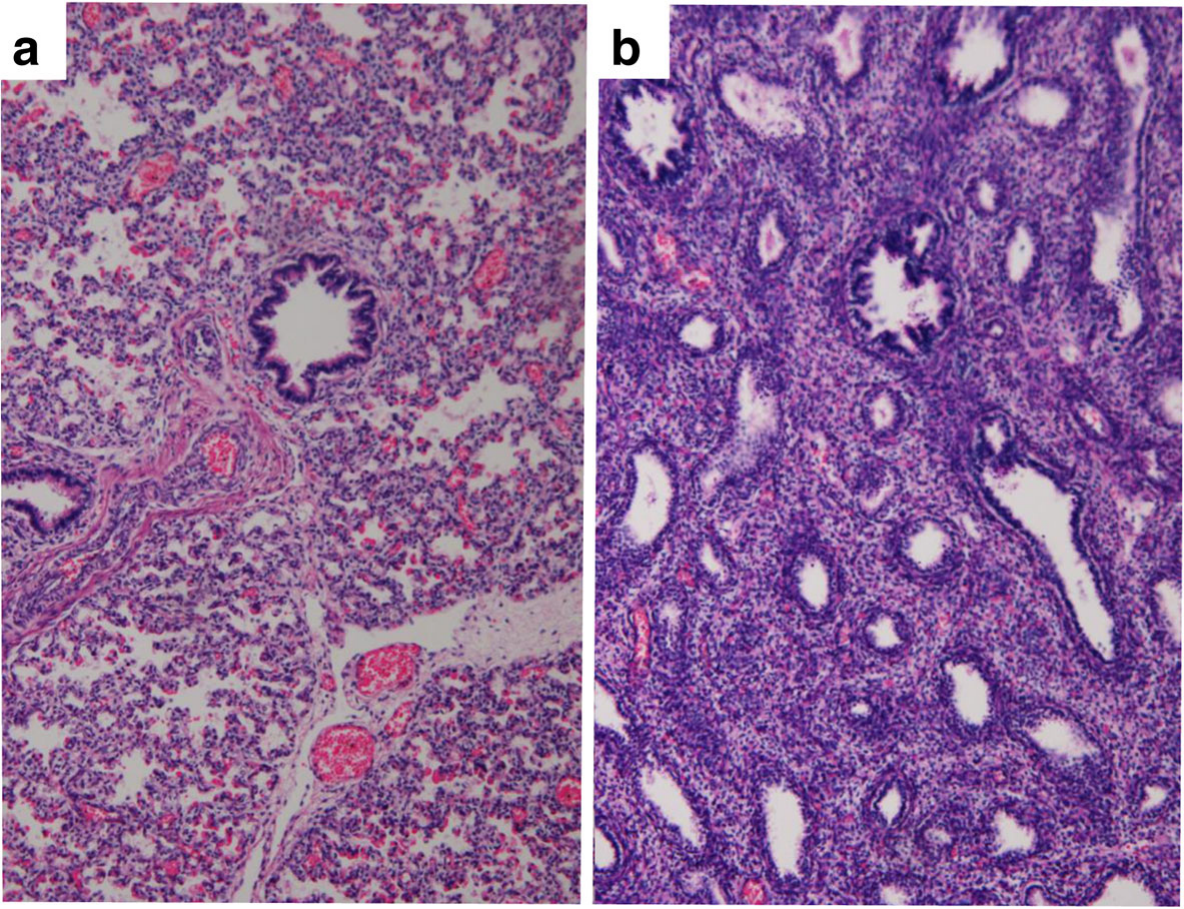
Microscopic images. **a** The lungs of the fetus are developed into the saccular stage (hematoxylin and eosin [HE] stain, × 100). **b** The lung seen in the epignathus is in the canalicular stage and is immature compared to the lungs of the fetus (HE stain, × 100)

Both the epignathus and intracranial lesion were composed of three germinal layers, with immature neuroepithelial tissue being the most predominant. Pulmonary components seen in the epignathus were equivalent to a canalicular stage of development, which was immature as compared to the saccular stage of the fetus’s internal lungs (Fig. 6). We, therefore, made the diagnosis of epignathus with intracranial extension, histologically showing immature teratoma.

As for the internal organs of the fetus, histological findings associated with hypoxia were seen. There was a great increase in weight seen in the liver and adrenal glands. Histologically, the liver showed congestion and marked hematopoiesis. Erythropoiesis usually diminishes and myelopoiesis becomes only faintly apparent in the liver by 28 weeks [6]; however, in our case, both erythropoiesis and myelopoiesis were marked in the liver as well as in the spleen. Furthermore, increased hemosiderin deposition was seen, not only within the periportal spaces, but also in surrounding hepatocytes. The cortices of both adrenal glands showed adenoid changes, with focal hemorrhaging. These findings in the liver and adrenal glands indicate hypoxic stress. The lungs weighed a little less than is typical with respect to the gestational week; however, histologically they had developed into the saccular stage. Because the kidneys and lungs were in the normal stage of development, the fetus could have swallowed some amniotic fluid in utero in spite of having the epignathus. Altogether, above-mentioned findings support the determination that an airway obstruction induced by the epignathus was the direct cause of death. The remainder of the detected organs (heart, thymus, gastrointestinal tract, gallbladder, pancreas, uterus, and ovaries) had developed sufficiently for the gestational week. The weight of the organs with means and standard deviations [7] are shown in Table 2. The placenta weighed 840 g and was histologically normal, except for the retroplacental hematoma, which was consistent with the clinical episode of placental abruption.

**Table 2:** Organ weights of the fetus with means and standard deviations [7]

	placenta [g]	body weight [g]	CRL [cm]	thymus [g]	heart [g]	lung (bilateral) [g]
Our case	840	1224–280 = 944	25	2.4	5.8	4.7 + 6.0 = 10.7
means and standard deviations [7]	240–380	836 ± 197	24.2 ± 2.5	2.3 ± 1.2	5.8 ± 1.9	22.1 ± 9.7
	spleen [g]	liver [g]	kidney (bilateral) [g]	adrenal gland (bilateral) [g]	brain [g]	
Our case	1.8	84.4	4.5 + 4.4 = 8.9	2.6 + 2.4 = 5.0	142.5	
means and standard deviations [7]	1.7 ± 1.0	35.1 ± 13.3	8.6 ± 3.0	2.5 ± 1.1	118 ± 21	

## Discussion and Conclusions

An epignathus is a rare congenital orofacial teratoma. In some studies, epignathi have been found to be associated with chromosomal abnormalities, such as duplication of 1q and 19p [8], ring X chromosome mosaicism [9], and the 49,XXXXY karyotype [10]. However, other studies have reported epignathi with no chromosomal abnormalities [11, 12]. The parents in our case requested that we not perform chromosome analysis; therefore, we were unable to establish a relationship between the epignathus and the chromosomal karyotype.

An epignathus frequently leads to fatal airway obstruction, so prenatal detection and early intervention to preserve ventilation is necessary. There have only been a few cases of epignathi that have been treated successfully following prenatal diagnosis and early interventions including surgery and chemotherapy. For example, Dapké et al. [11] reported a case with a three-year follow-up, in which prenatal detection was made at gestational week 27, and the fetus was born by cesarean section at gestational week 32. The patency of the airway was reestablished during delivery and the epignathus was removed immediately afterwards. Histologically, the epignathus was found to be a mature teratoma, and there was no intracranial extension.

Carvalho et al. [13] reported a seven-year follow-up case that also involved an epignathus determined to be a mature teratoma, with no intracranial extension. Carvalho’s case was exceptional for two reasons. First, the epignathus was not detected until the fetus was delivered vaginally at gestational week 40, due to inadequate prenatal care. Secondly, although the epignathus caused feeding problems, it did not obstruct the airway. Surgery to remove the epignathus was performed on postnatal day 14, and the residual tumor regressed after chemotherapy [13].

In both the cases reported by Dapké et al. [11] and Carvalho et al. [13], the epignathi were mature teratomas, and there was no intracranial extension.

An epignathus is infrequently associated with an intracranial lesion. In such cases, an intracranial teratoma needs to be differentiated from other intracranial tumors. Prenatal intracranial tumors can include a variety of neoplasms, such as teratomas, neuroepithelial tumors, and craniopharyngiomas [4]. It has been reported that prenatal intracranial teratomas typically result in poor prognoses [4, 5], and the clinical outcome of epignathus with intracranial extension is, to date, unclear. We were unable to find in the literature a single case of an epignathus with intracranial extension that was histologically determined to be an immature teratoma and was successfully treated with surgery or chemotherapy.

Some cases have been reported as epignathus with intracranial teratoma, and a common site of connection is the sella turcica [14], as seen in our case.

Thirteen cases of epignathus with intracranial tumor have been reported; the clinical and pathological summaries are shown in Table 3, and the earlier cases (1861–1963) are described in a report by Y. Hosoda [14].

**Table 3:** Clinical and pathological summary of our case and 13 previously reported cases of epignathus with intracranial tumor. Earlier cases (1861–1963) were presented in the report by Y. Hosoda [14]

Author	year	Mother	Autosite	Epignathus	Connection	Intracranial tumor
Wegelin [14]	1861	para 1	6 fetal mo., m.	8x10x6 cm. tridermal, feet with toes	sella turcica & crista galli	4 cysts in the base of skull
Breslau & Rindfleisch [14]	1864	28 y-o., para 1, polyhydroamnion	23–24 fetal w., f., B.W. 1548 g	fist-sized, mouth, eyes, extremities, Foetus in foetu	hypophyseal area, 8–10 cm stalk	tumor with extremities & umbilicus
Arnold [14]	1870	28 y-o., para 3	6 fetal mo., f., B.W. 1550 g	tridermal	sella turcica, 1 cm in diameter, consisted of glial tissue	5 cm in diameter, reached to right temporal scale, neuroglial
Müller [14]	1881	no information	full term, 6 days survived	23 cm in length, left oral cavity, broad based, ecto- & meso-derm	none	Walnut-sized, right middle cranial fossa, connective tissue & capillaries
Schükry [14]	1923	no information	7 fetal mo., f., cleft palate	chestnut-sized, pharynx, tridermal	sella turcica, firm fibrous tissue	right side of sella turcica, tridermal
Kraus [14]	1929	no information	premature, f., B.L. 42 cm	37 × 30 mm, intraoral, tridermal	craniopharyngeal canal, 7–8 mm in length, 4.5 mm in width	12x18x15 mm, sella turcica, tridermal
Ehrich [14]	1945	18 y-o., primipara, polyhydroamnion	5 fetal mo., still born, f., B.W. 1760 g, large head	5x8x4 cm, ecto- & meso-derm, pharynz	brain tissue in sphenoid bone	filling the cranial cavity, tridermal
Hosoda [14]	1963	26 y-o., gravidae iv, para 0	36 fetal w., f., B.W. 1950 g	6.5 × 3.5 × 2.0 cm, polypoid tumor hanging down from the upper lip, ecto- & meso-derm	no direct connection	anterior cranial fossa, 5 mm anterior to the sella turcica, spherical tumor, 4 × 3.5 × 2.8 cm, tridermal
Smith [15]	1993	30 y-o., para 3	29 fetal w., aborted., f., B.W. 1330 g	3.5 cm in diameter, oral tumor attached to the hard palate in the midline, tridermal	tumor was in continuity, via a narrow defect in the hard palate, sphenoid bone, third ventricle, extending to the lateral ventricles	central tumour mass separating the temporal lobes, which progected rostrally between dilated lateral ventricles, partly replacing the septum pellucidum
Smith [15]	1993	28 y-o., para 1, polyhydroamnion	18 fetal w., aborted., f.	2 cm in diameter, slightly gelatinous nodule protruding ftom the right upper lip and attached to the right maxilla, tridermal	narrow cord which traversed the nasal bones and entered the cranial cavity in the region of the pituitary fossa and direct communication with the mass in the frontal lobes.	partly cystic, lobular mass replacing the frontal. Predominantly neuroglial, with prominent ependymal rosettes
Johnston [10]	2007	no information	38 fetal w., m.,	4 × 5 cm, midline oropharyngeal mass protruding through a cleft deformity	none	anterior and middle cranial fossae
Calda [17]	2010	29 y-o., primigravida	20 fetal w., aborted., f., B.W. 310 g	2 cm in diameter, lobulated round vascular mass, visible trhough the open oral cavity, tridermal	none	13x10x7 mm, neuroepithelial intracranial cyst
Wang [12]	2017	31 y-o., primagravida	17 fetal w., aborted, m., 355 g	6.7 × 6.5 × 5.0 cm, protruding from the right maxillofacial region, tridermal	directly growing upward	middle and posterior cranial fossa, 3.5 cm in diameter, tridermal
Our case	2018	32 y-o., 3 gravida para 2	27 fetal w., stillborn, f., 1228 g	12x6x6 cm, 270 g, solid and multicystic tumor, based on the soft palate and protruding from mouth	the right side of the sella turcica, 2 mm in diameter	middle cranial fossa, capsuled tumor, 24 g, no direct invasion to the brain

Our case involved the largest epignathus with intracranial extension reported to date. Moreover, this epignathus was located at the base of the soft palate, and there was a direct invasion into the cranium through the right side of the sella turcica. The intracranial tumor was encapsulated and had not invaded the brain. Histologically, the epignathus and intracranial lesion were composed of cells originating from all three germinal layers. Importantly, the neural and pulmonary components were immature as compared to those of the fetal internal organs and brain tissue. Both shared the same histological features of immature teratomas, and, thus, we made the diagnosis of epignathus with intracranial extension.

Smith et al. [15] and Wang et al. [12] reported cases in which an epignathus had directly invaded the cranium. Within the literature review included in a study by Y, Hosoda [14], one case, reported by Ehrich et al., did not show a direct connection between the epignathus and intracranial lesion; however, brain tissue was seen in the sphenoid bone, which highly suggested a connection between the two lesions. On the other hand, Johnston et al. [16] and Calda et al. [17] reported cases where there were no direct connections between the epignathus and intracranial lesions. Calda et al. reported that an intracranial lesion had been suspected to be an expansion of an epignathus; however, a postmortem MRI and pathologic examination of the fetus confirmed an epignathus with bilateral ventricular dilatation, corpus callosum agenesis, and a neuroepithelial intracranial cyst [17]. While evaluation of an extension of an epignathus and intracranial lesion, including any invasion of the brain, is important, it is difficult to confirm this diagnosis prenatally. There have been cases reported in which an epignathus and intracranial tumor did not communicate directly, which promotes the necessity of careful evaluation when the fetus is suspected of having these lesions. Our case not only supports the findings that an epignathus can directly expand into the cranium, but also presents the possibility that an intracranial lesion can be encapsulated and does not necessarily invade the brain. We believe these rare but important findings will assist gynecologists, neurosurgeons, and pathologists in developing future therapeutic strategies when they are confronted with similar cases. Moreover, although our studied fetus was stillborn, this case provides hope for improved prenatal diagnosis, evaluation, and management of potential cases of epignathus with intracranial extension. However, further study of these rare cases is warranted.

## Abbreviations

EXIT: Ex utero intrapartum treatment
MRI: Magnetic resonance imaging
